# Creativity across the lifespan: changes with age and with dementia

**DOI:** 10.1186/s12877-023-03825-1

**Published:** 2023-03-22

**Authors:** Sabrina D. Ross, Thomas Lachmann, Saskia Jaarsveld, Steffi G. Riedel-Heller, Francisca S. Rodriguez

**Affiliations:** 1 German Center for Neurodegenerative Diseases (DZNE), RG Psychosocial Epidemiology & Public Health, Ellernholzstr. 1-2, 17394 Greifswald, Germany; 2 Cognitive and Developmental Psychology Unit, Center for Cognitive Science, University of Kaiserslautern-Landau, Kaiserslautern, Germany; 3 ³Centro de Investigación Nebrija en Cognición, Universidad Nebrija, Madrid, Spain; 4 Institute of Social Medicine, Occupational Health and Public Health (ISAP), Medical Faculty, University of Leipzig, Leipzig, Germany

**Keywords:** Creativity, Dementia, Age-related cognitive decline

## Abstract

**Background:**

It is well known that older age is associated with losses in cognitive functioning. Less is known about the extent to which creativity is changing with age or dementia. Aim of the current study was to gain more insights into psychometric aspects of creativity in younger and older people as well as people with dementia.

**Method:**

Our sample comprised three groups, (1) participants between age 18—30 years (*n* = 24), (2) participants 65 + years without cognitive impairment (*n* = 24), and (3) participants 65 + years with cognitive impairment / dementia (*n* = 23). Cognitive abilities were assessed via the *Standard Progressive Matrices Test* (SPM)*, Montreal Cognitive Assessment Test* (MoCa), and *Trail Making Test* (TMT). Creativity was assessed via the *Creative Reasoning Task* (CRT)*, Test of Creative Thinking-Drawing Production* (TCT-DP), and *Alternate Uses Task* (AUT).

**Results:**

Compared to younger people, older people scored significantly lower in only two out of eleven creativity sub-scores (one in the CRT and one in the TCT-DP). Performance in the SPM was significantly associated with these two sub-scores and age. Cognitively impaired older people had significantly lower scores in the creativity task AUT compared to cognitively healthy older people and younger people. The associations between MoCa and AUT scores were also significant.

**Conclusion:**

Creativity appears relatively stable in older age, with exception of those creativity skills that are affected by abstract reasoning (SPM), which appear susceptible to aging. As our findings suggest, cognitive impairment in older age might impair only some aspects of creativity with other creativity aspects being comparable to cognitively healthy people. The age-related and the cognitive status-related effects seem to be independent. The preserved creative abilities can be used in dementia care programs.

**Supplementary Information:**

The online version contains supplementary material available at 10.1186/s12877-023-03825-1.

## Introduction

Age-related changes occur not only in physical and functional abilities, but also affect our cognitive abilities. Many studies have shown that aging is associated with deterioration of performance in various cognitive abilities. This includes memory, logical thinking speed of processing, executive functions, working memory, and language [1–4]. This decline may be caused by atrophy in the cerebral cortex [5, 6], changes in executive function efficiency [7, 8], and slowing nerve impulses [9]. Age-related changes in another cognitive domain, namely creativity, is not well understood.

Creativity contributes to individual behavior and achievements and involves several complex cognitive processes. Creativity can be described as the ability to generate novel and original ideas within contextual constraints [10–12] and involves problem analysis, internal problem representation, selection of actions, organization of own cognitive resources, combining thinking strategies, and searching for alternative approaches. Researchers commonly divide creative thinking into two distinct processes: divergent and convergent thinking. Divergent thinking is a process of thinking in which multiple new solutions are possible [13, 14], whereas convergent thinking is the process of finding a specific solution to well-defined problem [13, 15].

Creativity and intelligence are positively correlated [16, 17]. While intelligence is considered to be a series of basic cognitive processes, creativity is required for solving complex tasks [17]. As research points out that cognitive performance tends to decrease with aging, one may conclude that creativity also decreases with higher age. Dietrich and Srinivasan [18] observed that the innovative creative phase of creative artists and scientists peaks before the age of forty [19]. They suggest that this is due to the neurodegenerative decline of the prefrontal cortex [18, 20], which leads to a decrease of cognitive and creative abilities. Further, in older age, many people develop dementia, a cognitive disorder characterized by progressive cognitive decline and abnormalities in the prefrontal cortex connectivity [21]. Hence, creativity might also be increasingly impaired with advancing dementia. Previous studies [22] have shown, on one hand, that people with dementia can still exhibit impressive creative abilities, and, on the other hand, that divergent thinking seems to decrease with dementia. However, only few studies have been conducted on this topic, most of them involving artists, and it is difficult to generalize findings.

A much larger number of studies exist on using arts and creativity to improve wellbeing of people with dementia. Several studies have shown that art-based therapy can be implemented well with people with dementia and offers a way to express their self and their emotions despite impaired language ability [22–25]. Findings indicate that art-based therapy can improve well-being and reduce depression and isolation, [22–25]. Although people with dementia often no longer draw pictures correctly, their creative abilities seem to be preserved [26]. While their semantic knowledge declines, their creativity seems to work. However, specific information on the psychometric aspects of changes of creativity with dementia are not yet known.

To fill the knowledge gap, the present study aims to gain a better understanding of the differences in creativity between people with and without dementia. Moreover, because it is not known how creativity changes in general across lifespan, we also investigate how creativity differs between younger and older people. Since previous research revealed that intelligence and creativity share the same cognitive faculty, we hypothesize that as cognition decreases with age, especially in terms of dementia, so does creativity, despite promising indications from few studies of creative abilities remaining unaffected in dementia stages.

## Methods

### Study design

We investigated the following three groups, (1) younger participants between 18—30 years of age, (2) participants 65 + years without cognitive impairment, and (3) 65 + years with cognitive impairment / dementia. Participants were recruited via flyers, general practitioners, and email distributors in Germany. Inclusion criteria were being (1) at least 18 years old, (2) having the capacity to provide informed consent (not delirious / no impaired consciousness), (3) not having test-relevant motor disorders, and (4) having sufficient visual and hearing abilities to ensure reliable test performance. Participants were excluded if they had any other neurological diseases (besides dementia) or insufficient physical abilities or psychological well-being to participate in the study, or if it was unclear if the person could effectively consent to participate in the study (e.g. cognitive status, German language skills). A total of *n* = 24 participants were ‘younger participants’ between 18—30 years of age. A total of *n* = 24 were participants 65 + years without cognitive impairment, and *n* = 23 participants were 65 + years with cognitive impairment/dementia. Cognitive impairment and/or dementia was assessed by self-reported health status and the Montreal Cognitive Assessment (MoCa) [27]. Participants who scored below 26 points in the MoCa were classified as having a cognitive impairment [27].

The study was approved by the ethics committees of the University of Greifswald (BB 012/20), the University of Leipzig (319/19-3 k) and the University of Kaiserslautern (mh/18/2019).

The procedure of the study started by checking the eligibility to participate according to the inclusion and exclusion criteria. Participants were informed about the details of the study and signed an informed consent. Then, demographic data were obtained (displayed in Table 1). Afterwards, a research team that was experienced and trained in data collection assessed cognitive performance and creativity. The study took place either in the participants' homes or in a research institute.

**Table 1:** Characteristics of the study participants

	**Younger (18 – 30 years) People (*n*** ** = 24)**	**Older (+ 65 years), Cognitive Healthy People (*n*** ** = 24)**	**Older (+ 65 years) Cognitive Impaired People (*n*** ** = 23)**
**Participants characteristics**	Frequency (n, %)	Frequency (n, %)	Frequency (n, %)
Age group			
18 – 30 years	24 (100%)	-	-
65 – 70 years	-	10 (41.67%)	2 (8.7%)
71 – 80 years	-	10 (41.67%)	11 (47.83%)
81 – 90 years	-	4 (16.66%)	9 (39.13%)
91 – 100 years	-	-	1 (4.34%)
Gender			
Female	16 (66.67%)	14 (58.33%)	15 (65.22%)
Male	8 (33.33%)	10 (41.67%)	8 (34.78%)
Education			
< 12 years	2 (8.33%)	1 (4.17%)	2 (8.7%)
≥ 12 years	22 (91.67%)	23 (95.83%)	21 (91.3%)
Marital status			
Solitarily	16 (66.67%)	6 (25%)	7 (30.43%)
In a partnership	8 (33.33%)	18 (75%)	16 (69.57%)
Diagnosed medical diseases			
No chronic diseases	21 (87.5%)	13 (54.17%)	11 (47.83%)
One chronic disease	3 (12.5%)	5 (20.83%)	10 (43.48%)
≥ 2 chronic diseases	-	6 (25%)	2 (8.69%)

*n* Number of participants in the corresponding group, *%* Frequency in the group in per cent

## Materials

### Cognitive performance

#### Montreal cognitive assessment

The cognitive status of the participants was assessed via the Montreal Cognitive Assessment (MoCa) test [27]. It is a short clinical test to assess with 30 questions various cognitive functions like attention, executive functioning, memory, language, visuospatial abilities, conceptual thinking, calculation, and orientation. Normal ageing participants score between 26 to 30 [27].

#### Standard progressive matrices test

The Standard Progressive Matrices Test (SPM) [28] is a non-verbal intelligence test. It assesses abstract reasoning [29], working memory [30], and convergent thinking in a well-defined problem space [31]. The SPM consists of 60 items, grouped into 5 sets (A, B, C, D, E) of which each has 12 items. The items are presented in a 1 × 1, 2 × 2, or 3 × 3 matrix, in which there is always an item missing in the lower right corner. Below the matrix, eight possible solutions are displayed. The participant is asked to complete the matrix by indicating the right solution. The items become progressively more difficult within and across sets. For each correctly solved problem, one point is awarded. A maximum of 60 points can be achieved.

#### Trail making test

Speed of information processing was measured using *Trail Making Test* (TMT) [32]. The TMT represents a trail-making test, in which where randomly arranged numbers from 1 to 90 must be connected in an ascending order. Participants are instructed to complete the TMT as quickly and accurately as possible. The TMT correlates (*r* = 0. 40 – 0.83) [33] with standard psychometric tests of intelligence (e.g. SPM) [34, 35] and is therefore regarded as an indicator for convergent thinking.

### Creativity

#### Creative reasoning task

The *Creative Reasoning Task* (CRT) captures cognitive thinking processes in an ill-defined problem space, in which both intelligent and creative abilities are required [31, 36, 37]. Participants are challenged to conceptualize a Raven matrix (similar to those in the SPM) that should be as original and as complex as possible. Participants can freely choose between different matrix types from the SPM: 1 × 1, 2 × 2, and 3 × 3 [36]. Created matrices are scored according to the scoring scheme by Jaarsveld, Lachmann [36]. In the current study, we used the *Relations* score (CRT-relations), which measures those creative processes that are supported by intelligence functioning in ill-defined problem space. It is calculated by evaluating the logical complexity of the matrix that the participants have produced. The sub-score CRT-relations is the sum of the values assigned to each component in a relationship with another component of the matrix that was created by the participant. The values represent the complexity of the relationships. Twelve relationship types were assessed (Idiosyncratic coherence, Jigsaw, Pattern completion, Iteration, Symmetry, Change, Increase, Succession, Indication of mathematical operations, Two values, Contrast, Groups of components, for further details see Jaarsveld et al., (2012)).

To assess the presented characteristics of the components, we also used a simple version of the *Components and Specifications* score [36] (CRT- CompandSpec): We determined whether the matrix components were figurative (coded 1) or geometrical (coded 2). All scores were evaluated in collaboration of two raters (SDR, FSR).

#### Test of creative thinking – drawing production

The *Test of Creative Thinking – Drawing Production (TCT-DP)* [38] is an instrument that evaluates a person’s creative potential in figure drawing. In this study, we administered form A, a drawing made up of a large frame with five simple line figures within its border and a sixth fragment outside the large frame. Participants were asked to complete the drawing. They were assured that there is no incorrect way to complete the drawing. The TCT-DP score (TCT-DP score) measures divergent thinking by evaluating the elaboration, number, originality and organization of ideas [39]. The score is composed of 13 sub-scores: (1) Continuation, (2) Completion, (3) New elements, (4) Connections Made with a Line, (5) Connections Made to Produce a Theme, (6) Dependent Figure-based Boundary Transgression, (7) Independent Figure-based Boundary Transgression, (8) Perspective, (9) Humor and Affectivity, (10) Unconventionality-a: any manipulation of the material; (11) Unconventionality-b: any surrealistic, fictitious and/or abstract elements or drawings; (12) Unconventionality-c: any usage of symbols or signs; (13) Unconventionality-d: unconventional use of given fragments which was combined to a *Total score* ranging from 0 – 66. The TCT-DP was evaluated by two independent raters (SDR, FSR; α = 0.97). An additional TCT-DP score was evaluated by the consensual assessment technique [40] with regard to the first subjective creative impression of the resulting drawing. This *Impression score* (TCT-DP imp) was rated by three independent raters (SDR, JSF, NZ) on a scale from 0 to 7: 0 = “not at all creative” to 7 = “extremely creative” (α = 0.90).

#### Alternate uses task

Divergent thinking was assessed using the *Alternate Uses Task* (AUT) [41]. Participants were asked to think of unusual, creative, and uncommon uses of a can, a paperclip, and a brick. The AUT was analysed based on Silvia [42], Silvia [43] and by using the consensual assessment technique [40]. First, *Fluency* (AUT fluency) was scored by counting the number of responses. Second, *Creativity* was scored by three independent raters (SDR, NZ, FSR) on a 5-point Likert scale ranging from 1 (not at all creative) to 5 (very creative) (α = 0.88, α = 0.82, and α = 0.75 for item 1, 2 and 3, respectively). The creativity scores of all generated ideas for an item were added up to the *Total creative score* (AUT score). A high correlation with the *Fluency* score does not support an independent interpretation of these scores [42–44]. In addition, an *Originality* (AUT origin) was calculated to measure the ability to produce ideas that are more original than the ideas of other participants. We assigned 1 point for responses given by > 2%—5% of the sample, 2 point for responses at least given by <  = 2% of the sample, and 0 points for responses mentioned by > 5% of the sample, respectively.

### Data analysis

To examine associations between cognitive performance and age, statistical relevant differences between younger (18—30 years) and older people (65 + years, cognitively healthy and cognitively impaired together) were estimated via the non-parametric Kruskal–Wallis test [45] in combination with the Dunn´s test [46] for pairwise multiple comparison. In addition, we ran linear regression modeling adjusted for confounder (gender, education, marital status, chronic diseases, and age groups).

To examine the association between creativity and age (younger vs. older (cognitively healthy and cognitively impaired together) people) as well as creativity and cognitive status (cognitively healthy vs. cognitively impaired), we ran the non-parametric Kruskal–Wallis test [45] in combination with the Dunn´s test [46] for pairwise multiple comparison, for each creativity task separately. Then, we conducted linear regression analysis comparing cognitively healthy with cognitively impaired participants, including age, gender, education, gender, marital status, and chronic diseases in the model. The confounder education was not included in the model for the CRT-sub-score CompandSpec since education explained 100% of the variance.

To examine to what extent cognition predicts creative thinking, we ran univariate and adjusted (gender, education, marital status, chronic diseases, and age groups) linear regression models, for each cognitive test separately.

All statistical analyses were conducted using STATA Version 15.0 and *p* < 0.05 as the significance level (two-tailed).

## Results

Characteristics of the participants are shown in Table 1. About two thirds of the participants were female. Only 7% had less than 12 years of education and 40.8% were single. Of the younger age group (*n* = 24), 87.5% had no chronic health condition. Of the cognitively healthy older age group (*n* = 24), 54.2% had no chronic health condition, and of the cognitively impaired older age group (*n* = 23), 47.8% had no chronic health condition.

### Associations between cognitive performance and age

Younger participants performed significantly better in the SPM and MoCa than older participants in general (cognitively healthy and cognitively impaired), but not in the TMT, as findings from the Kruskall-Wallis-Test (Supplementary file, Table S.1) and regression modelling (Table 2) indicate. Analysis regarding gender effects revealed the same results (even though the effects for TMT were not significant for men; Supplementary file, Table S.2).

**Table 2:** Estimates of the associations between Younger (age 18 – 30)/Older (age + 65, cognitively healthy and impaired) people and cognitive performance (MoCa, ZVT, and SPM)

	**MoCa**		**TMT**		**SPM**	
	β (95% Conf. Int)	p	β (95% Conf. Int)	p	β (95% Conf. Int)	p
Older (Ref: Younger)	1.38 (0.59 – 2.18)	0.001	-0.26 (-1.7 – 1.17)	0.723	1.9 (0.84 – 2.95)	0.001
Male (Ref: Female)	-0.08 (-0.26 – 0.10)	0.387	0.17 (-0.15 – 0.50)	0.296	0.03 (-0.21 – 0.27)	0.818
Education 12 + years (Ref: < 12 years)	0.68 (0.32 – 1.04)	0.000	0.14 (-0.67 – 0.69)	0.966	0.60 (0.13 – 1.08)	0.014
Living in partnership (Ref: solitarily)	-0.04 (-0.25 – 0.17)	0.720	0.06 (-0.33 – 0.45)	0.749	-0.05 (-0.33 – 0.24)	0.747
Chronic diseases (Ref: None)						
One chronic disease	0.13 (-0.08 – 0.35)	0.222	-0.08 (-0.46 – 0.30)	0.681	0.14 (-0.15 – 0.43)	0.337
≥ 2 chronic diseases	-0.31 (-0.61– -0.02)	0.037	-0.51 (-1.04 – 0.01)	0.054	0.18 (-0.21 – 0.56)	0.371
Age groups	-0.32 (-0.47 – -0.17)	< 0.001	0.13 (-0.14 – 0.40)	0.341	-0.51 (-0.71 – -0.32)	< 0.001

*MoCa* Montreal Cognitive Assessment, *Ref* reference category, *TMT* Trail Making Test, *SPM* Standard Progressive Matrices Test, *β (95% Conf. Int)* β with 95% Confidence Interval, *p *p– value

### Associations between creativity and age

**Fig. 1:**
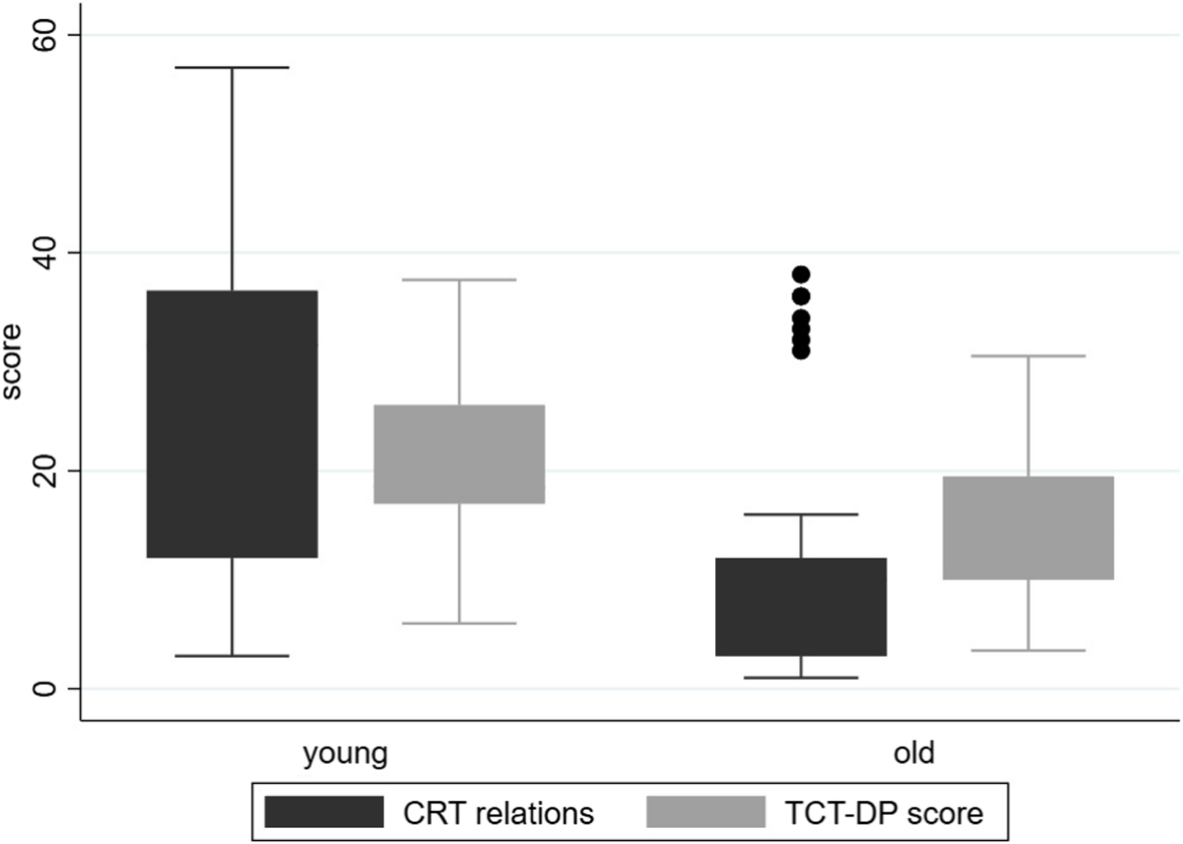
Means in the creativity task sub-scores CRT-relations and TCT-DP score for younger (age 18-30) and older (age 65 + years; cognitively healthy and impaired) people

Younger participants scored significantly higher than older participants (cognitively healthy and cognitively impaired) on two creativity scores, CRT relations and TCT-DP score. All other creativity scores being similar and non-significant between younger and older participants (Supplementary file, Table S.1; Fig. 1). Repeating the analysis treating cognitively healthy and cognitively impaired older adults as separate groups, we saw the same effects (Supplementary file, Table S.3). Adjusted linear regression analysis confirmed the effect (Table 3).

**Table 3:** Estimates of the associations between cognitive status (older, cognitively healthy and older, cognitive impaired) and creativity (CRT, TCT, and AUT)

	**CRT relations**		**CRT compandSpec**		**TCT-DP imp**		**TCT-DP score**		**AUT fluency**		**AUT score**		**AUT origin**	
	β (95% Conf. Int)	p	β (95% Conf. Int)	p	β (95% Conf. Int)	p	β (95% Conf. Int)	p	β (95% Conf. Int)	p	β (95% Conf. Int)	p	β (95% Conf. Int)	p
Cognitively impaired (Ref: healthy)	-0.32 (-0.87 – 0.23)	0.250	0.34 (-1.24 – 1.93)	0.672	-0.45 (-1.27 – 0.38)	0.283	-3.06 (-7.27 – 1.16)	0.152	-2.1 (-3.37 – -0.81)	0.002	-6.00 (-9.36 – -2.65)	0.001	-2.04 (-3.25 – -0.82)	0.001
Male (Ref: Female)	0.07 (-0.40 – 0.54)	0.764	-1.43 (-2.92 – 0.05)	0.058	-0.08 (-0.80 – 0.64)	0.828	-2.26 (-5.97 – 1.44)	0.227	0.33 (-0.8 – 1.46)	0.562	0.71 (-2.25 – 3.67)	0.635	0.26 (-0.81 – 1.33)	0.629
Education 12 + years (Ref: < 12 years)	-0.03 (-1.03 – 0.98)	0.958	-	-	-0.11 (-1.53 – 1.31)	0.880	1.73 (-5.56 – 9.03)	0.636	2.09 (-0.12 – 4.29)	0.063	4.81 (-0.98 – 10.6)	0.102	1.29 (-0.82 – 3.4)	0.227
Living in partnership (Ref: solitarily)	0.25 (-0.31 – 0.80)	0.373	0.75 (-0.89 – 2.39)	0.369	0.08 (-0.71 – 0.86)	0.845	1.14 (-2.88 – 5.16)	0.573	0.08 (-1.12 – 1.29)	0.891	0.16 (-3.00 – 3.32)	0.922	0.11 (-1.04 – 1.26)	0.850
Chronic diseases (Ref: None)														
One chronic disease	0.03 (-0.55 – 0.61)	0.922	-0.66 (-2.25 – 0.92)	0.411	-0.52 (-1.37 – 0.33)	0.228	-0.84 (-5.21 – 3.53)	0.703	-0.14 (-1.47 – 1.19)	0.831	-0.52 (-4.01 – 2.97)	0.768	-0.24 (-1.50 – 1.02)	0.703
≥ 2 chronic diseases	0.2 (-0.53 – 0.93)	0.584	-	-	0.04 (-1.11 – 1.19)	0.950	3.38 (-2.51 – 9.27)	0.256	0.17 (-1.61 – 1.95)	0.849	0.68 (-4 – 5.35)	0.773	0.04 (-1.67 – 1.75)	0.963
Age groups	-0.24 (-0.35 – 0.12)	< 0.001	0.3 (-0.04 – 0.63)	0.080	0.01 (-0.16 – 0.18)	0.927	-1.27 (-2.15 – -0.38)	0.006	-0.01 (-0.27 – 0.25)	0.939	-0.00 (-0.69 – 0.69)	0.999	0.16 (-0.1 – 0.41)	0.217

*CRT* *relations* Creative Reasoning Task – Relations, *CRT* *compandspec* Creative Reasoning Task – Components and Specifications, *TCT*—*DP* *imp* First impression mean score of the Test of Creative Thinking – Drawing Production, *TCT* – *DP* *score* Total score of the Test of Creative Thinking – Drawing Production, *AUT* *fluency* Mean fluency of generated number responses of the Alternate Uses Task, *AUT* *score* Mean creativity score of the Alternate Uses Task, *AUT* *origin* Statistical originality score of the Alternate Uses Task, *β (95% Conf. Int)* β with 95% Confidence Interval, *p *p – value, *Ref* Reference category

### Associations between creativity and cognitive status

**Fig. 2:**
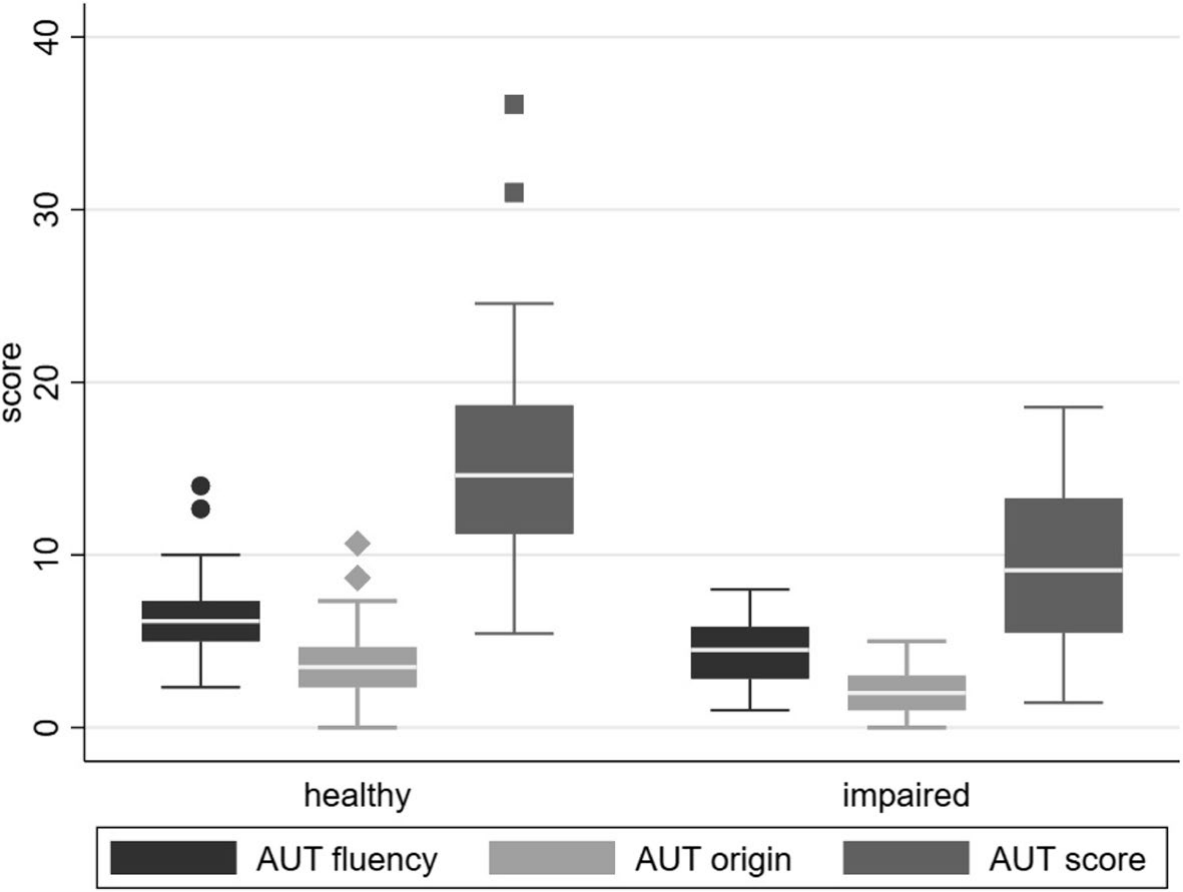
Means in the creative task AUT for older cognitively healthy and cognitively impaired people

Compared to the cognitive impaired, cognitive healthy older participants scored significantly higher in all creativity scores, except the CRT (Supplementary file, Table S.3). The TCT-DP score was the only score that differed significantly between all three groups, with cognitively impaired older adults having the lowest scores and younger participants having the highest scores. Linear regression analysis adjusted for confounders confirmed that cognitively impaired peopled had worse performance in the AUT (Fig. 2), but not in the other creativity tests (Table 3). Analysis regarding gender effects indicates a significant difference between cognitively healthy and cognitively impaired women in the TCT-DP, which remained non-significant in men (Supplementary file, Table S.4).

### Association between cognitive performance and creativity

In a final step, we estimated the extent to which cognitive abilities might affect creativity. Better performance in the Moca was significantly associated with higher scores in the TCT and AUT (Table 4). Better performance in the SPM was significantly associated with CRT relations, and the TCT scores. The TMT was not significantly associated with any creativity score (Table 4).

**Table 4:** Unadjusted and adjusted estimates on the association of cognitive performance on creativity scores

	**MoCa**		**TMT**		**SPM**	
	β (95% Conf. Int)	p	β (95% Conf. Int)	p	β (95% Conf. Int)	p
CRT relations						
unadjusted	0.10 (0.05 – 0.15)	< 0.001	0.00 (-0.00 – 0.00)	0.232	0.05 (0.03 – 0.07)	< 0.001
adjusted	0.07 (-0.00 – 0.13)	0.054	-0.00 (-0.00 – 0.00)	0.947	0.04 (0.01 – 0.06)	0.003
CRT compandspec						
unadjusted	-0.00 (-0.13 – 0.12)	0.942	0.00 (-0.00 – 0.01)	0.366	-0.05 (-0.1 – -0.00)	0.036
adjusted	0.01 (-0.23 – 0.25)	0.924	0.00 (-0.00 – 0.01)	0.549	-0.03 (-0.11 – 0.04)	0.420
TCT-DP imp						
unadjusted	0.08 (0.02 – 0.14)	0.006	-0.00 (-0.01 – 0.00)	0.085	0.03 (0.01 – 0.06)	0.004
adjusted	0.11 (0.04 – 0.19)	0.004	-0.00 (-0.01 – 0.00)	0.152	0.06 (0.02 – 0.09)	0.001
TCT-DP score						
unadjusted	0.66 (0.34 – 0.98)	< 0.001	-0.02 (-0.03 – 0.00)	0.015	0.34 (0.22 – 0.46)	< 0.001
adjusted	0.43 (0.03 – 0.83)	0.035	-0.01 (-0.03 – 0.01)	0.191	0.29 (0.10 – 0.47	0.003
AUT fluency						
unadjusted	0.19 (0.1 – 0.29)	< 0.001	-0.00 (-0.01 – 0.00)	0.132	0.06 (0.01 – 0.1)	0.008
adjusted	0.19 (0.07 – 0.32)	0.003	-0.00 (-0.01 – 0.00)	0.096	0.04 (-0.02 – 0.11)	0.171
AUT score						
unadjustied	0.53 (0.28 – 0.78)	< 0.001	-0.01 (-0.2 – 0.00)	0.067	0.16 (0.05 – 0.27)	0.005
adjusted	0.55 (0.22 – 0.88)	0.002	-0.01 (-0.03 – 0.00)	0.053	0.13 (-0.03 – 0.3)	0.114
AUT origin						
unadjusted	0.12 (0.03 – 0.22)	0.010	-0.00 (-0.01 – 0.00)	0.342	0.03 (-0.01 – 0.07)	0.102
adjusted	0.16 (0.04 – 0.28)	0.012	-0.00 (-0.01 – 0.00)	0.296	0.04 (-0.02 – 0.10)	0.152

*MoCa* Montreal Cognitive Assessment, *TMT* Trail MakingTest, *SPM* Standard Progressive Matrices Test, *β (95% Conf. Int)* β with 95% Confidence Interval, *p *p – value, *CRT* *relations* Creative Reasoning Task – Relations, *CRT* *compandspec* Creative Reasoning Task – Components and Specifications, *TCT*—*DP imp* First impression mean score of the Test of Creative Thinking – Drawing Production, *TCT – DP score* Total score of the Test of Creative Thinking – Drawing Production, *AUT fluency* Mean fluency of generated number responses of the Alternate Uses Task, *AUT score* Mean creativity score of the Alternate Uses Task, *AUT origin* Statistical originality score of the Alternate Uses Task

## Discussion

With this study, we wanted to gain a better understanding of how creativity evolves across the lifespan, especially in older age and with dementia. As expected, cognitive performance was significantly poorer in older age and with cognitive impairment. Creativity, on the other hand, did not show a general trend of decrease with age or cognitive status. We did observe age effects for two creativity sub-scores (CRT relations, TCT-DP score), both of which were lower with older age. Both scores were significantly associated with one cognitive performance test, the SPM, for which we also observed age effects. Other aspects of creativity seem to be relatively well preserved in older people. The main abilities required for the SPM, abstract reasoning and working memory, may contribute to creativity, an association between intelligence and creativity known from the literature [47]. The ability to be creative in an ill-defined problem-space (CRT relations, TCT-DP score) requires mental capacities, which people with higher intelligence possess and which decreases with age [48]. This may explain why we did not observe age-related effects for other creativity scores.

We also investigated how creativity differs between cognitively healthy and cognitively impaired older people. We found significant effects for one creativity test (AUT). The significant association of the cognitive performance MoCa scores with the AUT confirms this finding. Apart from this, people with dementia do not seem to have poorer creative abilities than cognitive healthy older people. This observation is supported by the fact that people with dementia can still paint pictures in art therapy, although the pictures have decreased complexity [26, 49]. The creativity test AUT is a verbal task so that it is possible that the worse performance among cognitively impaired people can be explained by the loss in verbal abilities that come with dementia. Another explanation could be that people with dementia just simply develop fewer alternative solutions [22].

Overall, fit seems that cognitively healthy older people and people with dementia have enough resources left to engage in creative activities. Research has shown that art-based therapy can help with behavioral symptoms such as depression and improve well-being of people with dementia [23, 25]. Whether and to what extent art-based therapy can protect against age-related cognitive decline or dementia is not yet clear as studies had only small sample sizes and low quality of study findings [50]. A recent study from Yu et al. [51] points out that art-based therapy can strengthen and enhance certain cognitive functions, such as immediate memory and working memory span, and increase in cortical thickness in the middle frontal gyrus [51]. Based on our findings, we believe that people with dementia might benefit from non-verbal creative activities in an ill-defined problem space, in which they can chose a logic or association freely rather than following strict rules. Further research is necessary to investigate that.

The current study has some limitations. Our sample has a high level of education and that the conclusions derived from our sample should be read with this restriction. Furthermore, cognitive status was assessed as self-reported or via the cognitive performance MoCa score. No clinical interview or other diagnostic measures were performed. Hence, our findings cannot be generalized for all dementia types. A neurological assessment with imaging data would provide more insights into how dementia pathology is related to creative potential.

## Conclusion

Across the lifespan, age-related changes occur and lead to a decrease in cognitive functioning, especially with dementia. Although cognition and creativity have functional overlaps in the brain, creativity seem to be relatively well preserved in older age and with dementia. Despite some losses in mental flexibility or number of solutions, our older participants came up with creative solutions. Thus, creativity can be incorporated into prevention and therapy programs to strength cognitive capacities or well-being. Programs such as these are urgently needed due to a growth in the prevalence of dementia [52].

## Supplementary Information

**Additional file 1.**

## Data Availability

The datasets generated and/or analysed during the current study are not publicly available due to privacy / ethical requirements but are available from the corresponding author on reasonable request.

## Abbreviations

SPM: Standard Progressive Matrices Test
MoCA: Montreal Cognitive Assessment
TMT: Trail Making Test
CRT: Creative Reasoning Task
CRT – relations: Creative Reasoning Task – Relations score
CRT – CompandSpec: Creative Reasoning Task – Score on components and specifications
TCT-DP: Test of Creative Thinking – Drawing Production
TCT-DP score: Total score of the Test of Creative Thinking – Drawing Production
TCT-DP imp: First impression mean score of the Test of Creative Thinking – Drawing Production
AUT: Alternate Uses Task
AUT fluency: Mean fluency of generated number responses of the Alternate Uses Task
AUT score: Mean creativity score of the Alternate Uses Task
AUT origin: Statistical originality score of the Alternate Uses Task
