# Correction to: Creativity across the lifespan: changes with age and with dementia

**DOI:** 10.1186/s12877-023-03964-5

**Published:** 2023-05-17

**Authors:** Sabrina D. Ross, Thomas Lachmann, Saskia Jaarsveld, Steffi G. Riedel-Heller, Francisca S. Rodriguez

**Affiliations:** 1 German Center for Neurodegenerative Diseases (DZNE), RG Psychosocial Epidemiology & Public Health, Ellernholzstr. 1-2, 17394 Greifswald, Germany; 2 Cognitive and Developmental Psychology Unit, Center for Cognitive Science, University of Kaiserslautern-Landau, Kaiserslautern, Germany; 3 Centro de Investigación Nebrija en Cognición, Universidad Nebrija, Madrid, Spain; 4 Institute of Social Medicine, Occupational Health and Public Health (ISAP), Medical Faculty, University of Leipzig, Leipzig, Germany

**BMC Geriatrics (2023) 23:160**

10.1186/s12877-023-03825-1

After publication of this article [1], the authors reported that the second author (Lachmann) is affiliated to affiliations 2 and 3 (instead of only 2); and the fourth author (Riedel-Heller) is affiliated to affiliation 4 (instead of 3).

The original article [1] has been corrected.
