# Supplementary material for: Creativity across the lifespan: changes with age and with dementia
Source: BMC Geriatr. 2023 Mar 22;23:160. doi: 10.1186/s12877-023-03825-1 (PMC10035174; doi:10.1186/s12877-023-03825-1)
Supplement: Supplementary file 1 — Additional file 1. [file 12877_2023_3825_MOESM1_ESM.docx]

**Supplementary File to the manuscript Creativity across the lifespan**

**Appendix**

**Table S.1** Differences in Cognition and Creativity scores between Younger (age 18-30 years) and Older (age 65+ years; cognitively healthy and impaired) people

| **Cognition** | **Mean (sd)** |  |  | **Kruskal Wallis Test** | | |
| --- | --- | --- | --- | --- | --- | --- |
|  | Total | **Younger People** (n=24) | **Older People** (n=47) | P | df | chi |
| MoCa | 25.59 (5.45) | 28.96 (1.55) | 23.87 (5.92) | <0.001 | 1 | 25.75 |
| TMT | 187.19 (127.75) | 152.16 (103.47) | 201.98 (135.04) | 0.069 | 1 | 3.29 |
| SPM | 32.66 (13.29) | 43.58 (6.89) | 27.09 (12.29) | <0.001 | 1 | 30.72 |
| **Creativity** | **Mean (sd)** |  |  | **Kruskal Wallis Test** | | |
|  | Total | **Younger People** (n=24) | **Older People** (n=47) | P | df | chi |
| CRT relations | 18.52 (14.82) | 28.63 (14.68) | 12.31 (11.13) | <0.001 | 1 | 14.77 |
| TCT - DP imp | 2.69 (1.37) | 2.88 (1.50) | 2.58 (1.3) | 0.458 | 1 | 0.55 |
| TCT - DP score | 17.11 (8.04) | 21.58 (8.19) | 14.78 (6.97) | 0.001 | 1 | 11.79 |
| AUT fluency | 5.7 (2.39) | 6.25 (2.52) | 5.41 (2.3) | 0.274 | 1 | 1.12 |
| AUT score | 13.16 (6.33) | 14.53 (6.94) | 12.45 (5.95) | 0.407 | 1 | 0.69 |
| AUT origin | 3.11 (2.21) | 3.1 (2.44) | 3.12 (2.12) | 0.614 | 1 | 0.25 |
|  | **N (%)** |  |  | **Pearson's chi-squared Test** | | |
|  | Total | **Younger People** (n=24) | **Older People** (n=47) | X^2^ | P | |
| CRT compandspec | 62 (100 %) |  |  | 2.73 | 0.098 | |
| fig | 15 (23.81 %) | 3 (12.5 %) | 12 (23.81 %) |  |  | |
| geom | 48 (76.19 %) | 21 (87.5 %) | 27 (76.19 %) |  |  | |

*Note*: sd = Standard Deviation; n = Number of participants in the corresponding group; P = p - value; df = Degree of Freedom; Chi = Chi square; X^2^  = Pearson chi-squared; MoCa = Montreal Cognitive Assessment; TMT = Trail Making Test; SPM = Standard Progressive Matrices Test; CRT relations = Creative Reasoning Task – Relations score; TCT - DP imp = First impression mean score of the Test of Creative Thinking – Drawing Production; TCT – DP score = Total score of the Test of Creative Thinking – Drawing Production; AUT fluency = Mean fluency of generated number responses of the Alternate Uses Task; AUT score = Mean creativity score of the Alternate Uses Task; AUT origin = Statistical originality score of the Alternate Uses Task; CRT compandspec = Creative Reasoning Task – Score on components and specifications; fig = Figural; geom = Geometrical

**Table S.2** Cognition and Creativity between younger (age 18-30) and older (age 65+ years; cognitive healthy and impaired) people with gender effects

| **Cognition** | **Mean (sd)** | |  | |  | | **Kruskal Wallis Test** | | | | | |
| --- | --- | --- | --- | --- | --- | --- | --- | --- | --- | --- | --- | --- |
|  | Total | | **Younger People** (n=24) | | **Older People** (n=47) | | P | | df | | chi | |
|  |  | | **f** | **m** | **f** | **m** | f | m | f | m | f | m |
| MoCa | 25.59 (5.45) | | 29.38 (0.62) | 28.13 (2.42) | 23.72 (6.55) | 24.11 (4.90) | <0.001 | 0.015 | 1 | 1 | 19.24 | 5.98 |
| TMT | 187.19 (127.75) | | 123 (36.10) | 215.33 (168.58) | 174.89 (82.91) | 246.59 (187.47) | 0.047 | 0.726 | 1 | 1 | 3.96 | 0.12 |
| SPM | 32.66 (13.29) | | 43.31 (5.84) | 44.13 (9.08) | 26.03 (13.12) | 28.78 (10.98) | <0.001 | 0.003 | 1 | 1 | 20.19 | 9.17 |
| **Creativity** | **Mean (sd)** | |  | |  | | **Kruskal Wallis Test** | | | | | |
|  | Total | | **Younger People** (n=24) | | **Older People** (n=47) | | P | | df | | chi | |
|  |  | | **f** | **m** | **f** | **m** | f | m | f | m | f | m |
| CRT relations | 18.52 (14.82) | | 26.31 (13.5) | 33.25 (16.74) | 12.27 (10.93) | 12.35 (11.73) | 0.006 | 0.006 | 1 | 1 | 7.56 | 7.5 |
| TCT - DP imp | 2.69 (1.37) | | 3.25 (1.6) | 2.13 (0.97) | 2.4 (1.27) | 2.85 (1.34) | 0.092 | 0.157 | 1 | 1 | 2.83 | 2.01 |
| TCT - DP score | 17.11 (8.04) | | 23.13 (9.5) | 18.5 (3.3) | 15.09 (7.08) | 14.31 (6.98) | 0.005 | 0.046 | 1 | 1 | 7.74 | 4.00 |
| AUT fluency | 5.7 (2.39) | | 6.17 (2.89) | 6.42 (1.72) | 5.18 (1.97) | 5.78 (2.75) | 0.414 | 0.637 | 1 | 1 | 0.67 | 0.22 |
| AUT score | 13.16 (6.33) | | 14.55 (7.92) | 14.49 (4.87) | 11.87 (4.97) | 13.36 (7.28) | 0.464 | 1.000 | 1 | 1 | 0.54 | 0.00 |
| AUT origin | 3.11 (2.21) | | 2.94 (2.64) | 3.42 (2.1) | 3.01 (2.11) | 3.3 (2.17) | 0.635 | 0.868 | 1 | 1 | 0.23 | 0.03 |
|  | **N (%)** | |  | |  | | **Pearson's chi-squared test** | | | | | |
|  | Total | | **Younger People** (n=24) | | **Older People** (n=47) | | X^2^ | | P | | | |
|  | f | m | **f** | **m** | **f** | **m** | f | m | f | | m | |
| CRT compandspec | 38 (100 %) | 25 (100%) |  |  |  |  | 1.4 | 2.24 | 0.237 | | 0.134 | |
| fig | 11 (28.95 % | 4 (16 %) | 3 (27.27 %) | 0 (0 %) | 8 (36.36 %) | 4 (23.53 %) |  |  |  | |  | |
| geom | 27 (71.05 %) | 21 (84 %) | 13 (81.25 %) | 8 (100 %) | 14 (63.64%) | 13 (76.47 %) |  |  |  | |  | |

*Note*: sd = Standard Deviation; n = Number of participants in the corresponding group; P = p - value; df = Degree of Freedom; Chi = Chi square; X^2^  = Pearson chi-squared; f = female; m = male; MoCa = Montreal Cognitive Assessment; TMT = Trail MakingTest; SPM = Standard Progressive Matrices Test; CRT relations = Creative Reasoning Task – Relations; TCT - DP imp = First impression mean score of the Test of Creative Thinking – Drawing Production; TCT – DP score = Total score of the Test of Creative Thinking – Drawing Production; AUT fluency = Mean fluency of generated number responses of the Alternate Uses Task; AUT score = Mean creativity score of the Alternate Uses Task; AUT origin = Statistical originality score of the Alternate Uses Task; CRT compandspec = Creative Reasoning Task – Components and Specifications; fig = Figural; geom = Geometrical

**Table S.3** Differences in Cognition and Creativity scores between Groups

| **Creativity** | **Mean (sd)** |  |  |  | **Kruskal Wallis Test** | | | **Dunn´s Test** | | | |
| --- | --- | --- | --- | --- | --- | --- | --- | --- | --- | --- | --- |
|  | Total | **Younger People** (n=24) | **Older, Cognitive Healthy People** (n=24) | **Older, Cognitive Impaired People** (n=23) | P | df | chi | P (Y:OU) | | P (Y:OI) | P (OU:OI) |
| CRT relations | 18.52 (14.82) | 28.62 (14.67) | 13.83 (11.38) | 10.13 (10.73) | <0.001 | 2 | 15.98 | 0.002 | | <0.001 | 0.134 |
| TCT - DP imp | 2.69 (1.37) | 2.88 (1.5) | 2.89 (1.31) | 2.24 (1.24) | 0.142 | 2 | 3.907 | 0.402 | | 0.055 | 0.033 |
| TCT - DP score | 17.11 (8.04) | 21.58 (8.19) | 17.21 (6.55) | 12.14 (6.57) | <0.001 | 2 | 18.86 | 0.045 | | <0.001 | 0.004 |
| AUT fluency | 5.7 (2.39) | 6.25 (2.52) | 6.64 (1.96) | 4.08 (1.87) | <0.001 | 2 | 16.84 | 0.163 | | 0.001 | <0.001 |
| AUT score | 13.16 (6.33) | 14.53 (6.94) | 16.00 (4.75) | 8.58 (4.58) | <0.001 | 2 | 20.90 | 0.070 | | 0.001 | <0.001 |
| AUT origin | 3.11 (2.21) | 3.1 (2.44) | 4.24 (1.99) | 1.96 (1.56) | 0.001 | 2 | 15.18 | 0.009 | | 0.061 | <0.001 |
|  | **N (%)** |  |  |  | **Pearson's chi-squared Test** | | | | | | |
|  | Total | **Younger People** (n=24) | **Older, Cognitive Healthy People** (n=24) | **Older, Cognitive Impaired People** (n=23) | X^2^ | | | | P | | |
| CRT compandspec | 63 (100 %) |  |  |  | 3.41 | | | | 0.182 | | |
| fig | 15 (23.81 %) | 3 (12.5 %) | 6 (26.09 %) | 6 (37.5 %) |  | | | |  | | |
| geom | 48 (76. 19) | 21 (87.5 %) | 17 (73.91 %) | 10 (62.5) |  | | | |  | | |

*Note*: sd = Standard Deviation; P = p - value; df = Degree of Freedom; Chi = Chi square; X^2^  = Pearson chi-squared; P (Y:OU) = p – value of the Dunn´s test when comparing younger to older, cognitive healthy participants; P (Y:OI) = p – value of the Dunn´s test when comparing younger to older, cognitive impaired participants; P (OU:OI) = p – value of the Dunn´s test when comparing older, cognitive healthy to older, cognitive impaired participants; MoCa = Montreal Cognitive Assessment; TMT = Trail Making Test; SPM = Standard Progressive Matrices Test; CRT relations = Creative Reasoning Task – Relations; TCT - DP imp = First impression mean score of the Test of Creative Thinking – Drawing Production; TCT – DP score = Total score of the Test of Creative Thinking – Drawing Production; AUT fluency = Mean fluency of generated number responses of the Alternate Uses Task; AUT score = Mean creativity score of the Alternate Uses Task; AUT origin = Statistical originality score of the Alternate Uses Task; CRT compandspec = Creative Reasoning Task – Components and Specifications; fig = Figural; geom = Geometrical

**Table S.4** Cognition and Creativity between Groups with gender effects

| **Cognition** | **Mean (sd)** | |  | |  | |  | | **Kruskal Wallis Test** | | | | | | **Dunn´s Test** | | | | | |
| --- | --- | --- | --- | --- | --- | --- | --- | --- | --- | --- | --- | --- | --- | --- | --- | --- | --- | --- | --- | --- |
|  | Total | | **Younger People** (n=24) | | **Older, Cognitive Healthy People** (n=24) | | **Older, Cognitive Impaired People** (n=23) | | P | | df | | chi | | P (Y:OU) | | P (Y:OI) | | P (OU:OI) | |
|  |  | | **f** | **m** | **f** | **m** | **f** | **m** | f | m | f | m | f | m | f | m | f | m | f | m |
| MoCa | 25.59 (5.45) | | 29.38 (0.62) | 28.13 (2.42) | 28.14 (1.29) | 27 (1.41) | 19.6 (6.82) | 20.5 (5.37) | <0.001 | <0.001 | 2 | 2 | 32.63 | 15.76 | 0.033 | 0.210 | <0.001 | <0.001 | <0.001 | 0.001 |
| TMT | 187.19 (127.75) | | 123 (36.10) | 215.33 (168.58) | 132.14 (38.26) | 185.1 (106.45) | 217.64 (94.23) | 334.43 (247.84) | 0.002 | 0.347 | 2 | 2 | 12.48 | 2.12 | 0.382 | 0.408 | 0.001 | 0.150 | 0.002 | 0.079 |
| SPM | 32.66 (13.29) | | 43.31 (5.84) | 44.13 (9.08) | 33.71 (8.04) | 32.9 (8.05) | 18.87 (13.04) | 23.63 (12.45) | <0.001 | 0.004 | 2 | 2 | 25.31 | 10.99 | 0.004 | 0.017 | <0.001 | 0.001 | 0.012 | 0.088 |
| **Creativity** | **Mean (sd)** | |  | |  | |  | | **Kruskal Wallis Test** | | | | | | **Dunn´s Test** | | | | | |
|  | Total | | **Younger People** (n=24) | | **Older, Cognitive Healthy People** (n=24) | | **Older, Cognitive Impaired People** (n=23) | | P | | df | | chi | | P (Y:OU) | | P (Y:OI) | | P (OU:OI) | |
|  |  | | **f** | **m** | **f** | **m** | **f** | **m** | f | m | f | m | f | m | f | m | f | m | f | m |
| CRT relations | 18.52 (14.82) | | 26.31 (13.5) | 33.25 (16.74) | 11.69 (9.75) | 16.6 (13.23) | 13.11 (13.02) | 6.29 (5.59) | 0.023 | 0.008 | 2 | 2 | 7.56 | 9.70 | 0.007 | 0.032 | 0.016 | <0.001 | 0.476 | 0.068 |
| TCT - DP imp | 2.69 (1.37) | | 3.25 (1.60) | 2.13 (0.97) | 2.9 (1.30) | 2.87 (1.38) | 1.9 (1.10) | 2.83 (1.38) | 0.030 | 0.365 | 2 | 2 | 7.02 | 2.01 | 0.349 | 0.094 | 0.006 | 0.121 | 0.020 | 0.467 |
| TCT - DP score | 17.11 (8.04) | | 23.13 (9.50) | 18.5 (3.30) | 18.14 (7.33) | 15.9 (5.36) | 12.04 (5.50) | 12.31 (8.55) | 0.002 | 0.056 | 2 | 2 | 12.54 | 5.78 | 0.106 | 0.115 | <0.001 | 0.008 | 0.014 | 0.091 |
| AUT fluency | 5.70 (2.39) | | 6.17 (2.89) | 6.42 (1.72) | 6.19 (1.78) | 7.27 (2.12) | 4.17 (1.64) | 3.92 (2.32) | 0.038 | 0.019 | 2 | 2 | 6.53 | 7.92 | 0.291 | 0.207 | 0.025 | 0.029 | 0.008 | 0.003 |
| AUT score | 13.16 (6.33) | | 14.55 (7.92) | 14.49 (4.87) | 14.83 (4.13) | 17.64 (5.27) | 8.91 (3.93) | 8.00 (5.81) | 0.008 | 0.007 | 2 | 2 | 9.63 | 10.00 | 0.176 | 0.078 | 0.015 | 0.048 | 0.001 | 0.001 |
| AUT origin | 3.11 (2.21) | | 2.94 (2.64) | 3.42 (2.10) | 3.86 (2.25) | 4.77 (1.52) | 2.22 (1.69) | 1.46 (1.25) | 0.113 | 0.001 | 2 | 2 | 4.36 | 14.26 | 0.070 | 0.034 | 0.272 | 0.032 | 0.021 | <0.001 |
|  | **N (%)** | |  | |  | |  | | **Pearson's chi-squared test** | | | | | | | | | | | |
|  | Total | | **Younger People** (n=24) | | **Older, Cognitive Healthy People** (n=24) | | **Older, Cognitive Impaired People** (n=23) | | X^2^ | | | | | | P | | | | | |
|  | f | m | **f** | **m** | **f** | **m** | **f** | **m** | f | | | m | | | f | | | m | | |
| CRT compandspec | 38 (100 %) | 25 (100 %) |  |  |  |  |  |  | 1.88 | | | 2.47 | | | 0.390 | | | 0.291 | | |
| fig | 11 (28.95 %) | 4 (16 %) | 3 (18.75 %) | 0 (0 %) | 4 (30.77 %) | 2 (20 %) | 4 (36.36 %) | 2 (28.57 %) |  | | |  | | |  | | | | | |
| geom | 27 (71.05 %) | 21 (84 %) | 13 (81.25 %) | 8 (100 %) | 9 (69.23 %) | 8 (80 %) | 5 (55.56 %) | 5 (71.43 %) |  | | |  | | |  | | | | | |

*Note*: sd = Standard Deviation; P = p - value; df = Degree of Freedom; Chi = Chi square; P (Y:OU) = p – value of the Dunn´s test when comparing younger to older, cognitive healthy people; P (Y:OI) = p – value of the Dunn´s test when comparing younger to older, cognitive impaired people; P (OU:OI) = p – value of the Dunn´s test when comparing older, cognitive healthy to older, cognitive impaired people; X^2^  = Pearson chi-squared; MoCa = Montreal Cognitive Assessment; TMT = Trail Making Test; SPM = Standard Progressive Matrices Test; CRT relations = Creative Reasoning Task – Relations; TCT - DP imp = First impression mean score of the Test of Creative Thinking – Drawing Production; TCT – DP score = Total score of the Test of Creative Thinking – Drawing Production; AUT fluency = Mean fluency of generated number responses of the Alternate Uses Task; AUT score = Mean creativity score of the Alternate Uses Task; AUT origin = Statistical originality score of the Alternate Uses Task; CRT compandspec = Creative Reasoning Task – Components and Specifications; fig = Figural; geom = Geometrical
